# Simple 3D culture of dissociated kidney mesenchyme mimics nephron progenitor niche and facilitates nephrogenesis Wnt-independently

**DOI:** 10.1038/s41598-019-49526-x

**Published:** 2019-09-17

**Authors:** Arvydas Dapkunas, Ville Rantanen, Yujuan Gui, Maciej Lalowski, Kirsi Sainio, Satu Kuure, Hannu Sariola

**Affiliations:** 1 Biochemistry and Developmental Biology, Faculty of Medicine, University of Helsinki, FI-00014 Helsinki, Finland; 2 Meilahti Clinical Proteomics Core Facility, Helsinki Institute of Life Science, University of Helsinki, FI-00014 Helsinki, Finland; 3 Genome-Scale Biology Research Program, Faculty of Medicine, University of Helsinki, FI-00014 Helsinki, Finland; 4 Institute of Biotechnology, Helsinki Institute of Life Science, University of Helsinki, FI-00014 Helsinki, Finland; 5 Stem Cells and Metabolism Research Program, Faculty of Medicine, University of Helsinki, FI-00014 Helsinki, Finland; 6 GM-unit, Laboratory Animal Centre, Helsinki Institute of Life Science, University of Helsinki, FI-00014 Helsinki, Finland

**Keywords:** Differentiation, Stem-cell niche

## Abstract

Kidney mesenchyme (KM) and nephron progenitors (NPs) depend on WNT activity, and their culture *in vitro* requires extensive repertoire of recombinant proteins and chemicals. Here we established a robust, simple culture of mouse KM using a combination of 3D Matrigel and growth media supplemented with Fibroblast Growth Factor 2 (FGF2) and Src inhibitor PP2. This allows dissociated KM to spontaneously self-organize into spheres. To reassess the requirement of WNT activity in KM self-organization and NPs maintenance, cells were cultured with short pulse of high-dose GSK3β inhibitor BIO, on a constant low-dose or without BIO. Robust proliferation at 48 hours and differentiation at 1 week were observed in cultures with high BIO pulse. Importantly, dissociated KM cultured without BIO, similarly to that exposed to constant low dose of BIO, maintained NPs up to one week and spontaneously differentiated into nephron tubules at 3 weeks of culture. Our results show that KM is maintained and induced to differentiate in a simple culture system. They also imply that GSK3β/WNT-independent pathways contribute to the maintenance and induction of mouse KM. The robust and easy 3D culture enables further characterization of NPs, and may facilitate disease modeling when applied to human cells.

## Introduction

The mammalian kidney develops from the intermediate mesoderm through reciprocal interactions of the ureteric bud (UB) and kidney mesenchyme (KM) known also as metanephric mesenchyme^1^. Nephrons, the functional units of the kidney that filter the waste products from the blood, form by orchestrated differentiation of the nephron progenitor cells (NPs) located in close proximity to UB tips. Mesenchymal NPs are first induced to aggregate and form renal vesicle, which then fully epithelializes via pretubular aggregate, comma- and S-shaped bodies^1^. It has been demonstrated that WNT pathway is a key paracrine signaling for maintenance of nephron progenitors and their timely differentiation into nephron epithelium^2,3^. *Wnt11* expressed by UB tips facilitates UB branching via positively reinforcing glial cell line derived growth factor (GDNF)/rearranged in transformation (RET) signaling, and participates in NPs maintenance^3,4^. *Wnt9b*, expressed by the UB trunk and to lesser extent in tips, maintains and induces SIX2-expressing NPs for differentiation^5–7^. In the differentiating nephron structures Wnt4 acting cell-autonomously is needed for completion of mesenchyme-to- epithelium transition (MET)^8^. Both canonical pathway involving Glycogen Synthase Kinase 3 Beta (GSK3β) inhibition and leading to β-catenin stabilization, as well as non-canonical pathway mediate WNT functions in self-renewal and nephrogenesis^7,9^.

Organotypic cultures, such as the transfilter system and explants of the whole kidney anlagen, have profoundly deepened our knowledge of the developmental mechanisms of kidney development^10^. However, it has been shown that when the KM is cultured in isolation, it stops proliferating, does not differentiate and decays in a few days^10,11^. First attempts towards the long-term KM *in vitro* culture were undertaken by dissociating and reaggregating whole embryonic kidneys where UB cells were present^12^. A variety of factors required for the maintenance, proliferation, and differentiation of the KM has been discovered^13–16^. Recently, the maintenance and propagation of purified nephrogenic progenitor cells was achieved^17,18^. Although efficient in maintaining the purified SIX2^+^ NPs, these cultures exclude stromal niche cells. In addition, the maintenance protocols are tedious, depend on complex procedures and require multiple synthetic agents and growth factors.

The majority of renal cell cultures rely on 2D monolayers^19–22^ that do not accurately model the 3D architecture of the tissue. The 3D architecture and cell-cell contacts are essential for propagation SIX2^+^ NPs *in vitro* both in isolation and when pluripotent stem cells are differentiated into kidney organoids^17,23^.

Until recently, the induction of an isolated kidney mesenchyme for differentiation was based on its recombination with the UB or heterologous inducing tissues such as embryonic spinal cord^21,24,25^. The GSK3β inhibitor 6-bromoindirubin-3-oxime (BIO) has been shown to induce differentiation of isolated rat and mouse mesenchyme via the canonical WNT signaling pathway^9^. Chemical induction with GSK3β inhibitors has been utilized not only for kidney explants but also for generating organoids from hiPSC that show nephron marker patterns typical for that of nephrogenesis *in vivo*^26^.

Here we describe a robust, simple culture of mouse KM using a combination of 3D Matrigel and growth media supplemented with FGF2 and PP2. We show that this method facilitates dissociated KM to self-organize into spheres where also renal stromal cells are present. We further demonstrate that NPs and stromal niche propagate for a week, and thereafter NPs undergo spontaneous MET to form nephron tubules without additional WNT activation. Our results imply that GSK3β/WNT-independent pathways complement regulation of self-renewal and differentiation decisions in mouse KM.

## Results

### Self-organization and formation of metanephric niche

**Figure 1:**
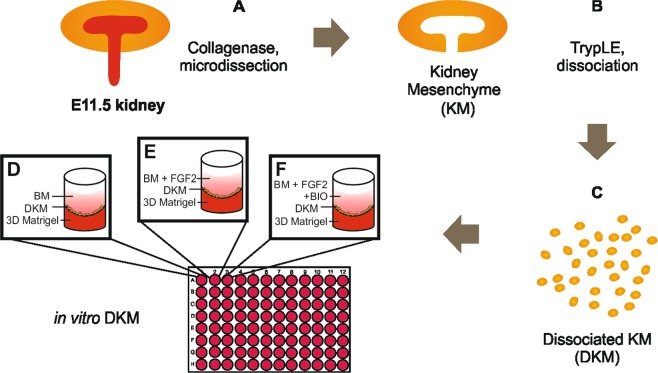
Schematic representation of the 3D culture method of dissociated kidney mesenchyme. (**A**) E11.5 embryonic mouse kidneys are isolated and subjected to collagenase IV treatment after which the KM is separated from the epithelial ureteric bud (UB) by manual microdissection. (**B**) Dissociation of KM into single cells assisted by TrypLE enzymatic digestion, resulting in single cell suspension (dissociated KM). (**C**) After pelleting the cells with centrifugation, the dissociated KM is resuspended into Basic Media (BM). Dissociated KM cells are then plated on top of the 3D Matrigel submerged in either (**D**) BM, (**E**) BM + 50 ng/ml FGF2 or (**F**) BM + 50 ng/ml FGF2 and pulsed with 10 µM BIO for 1 hour. In some experiments BIO pulse is replaced by constant BIO application at 50 nM concentration.

**Figure 2:**
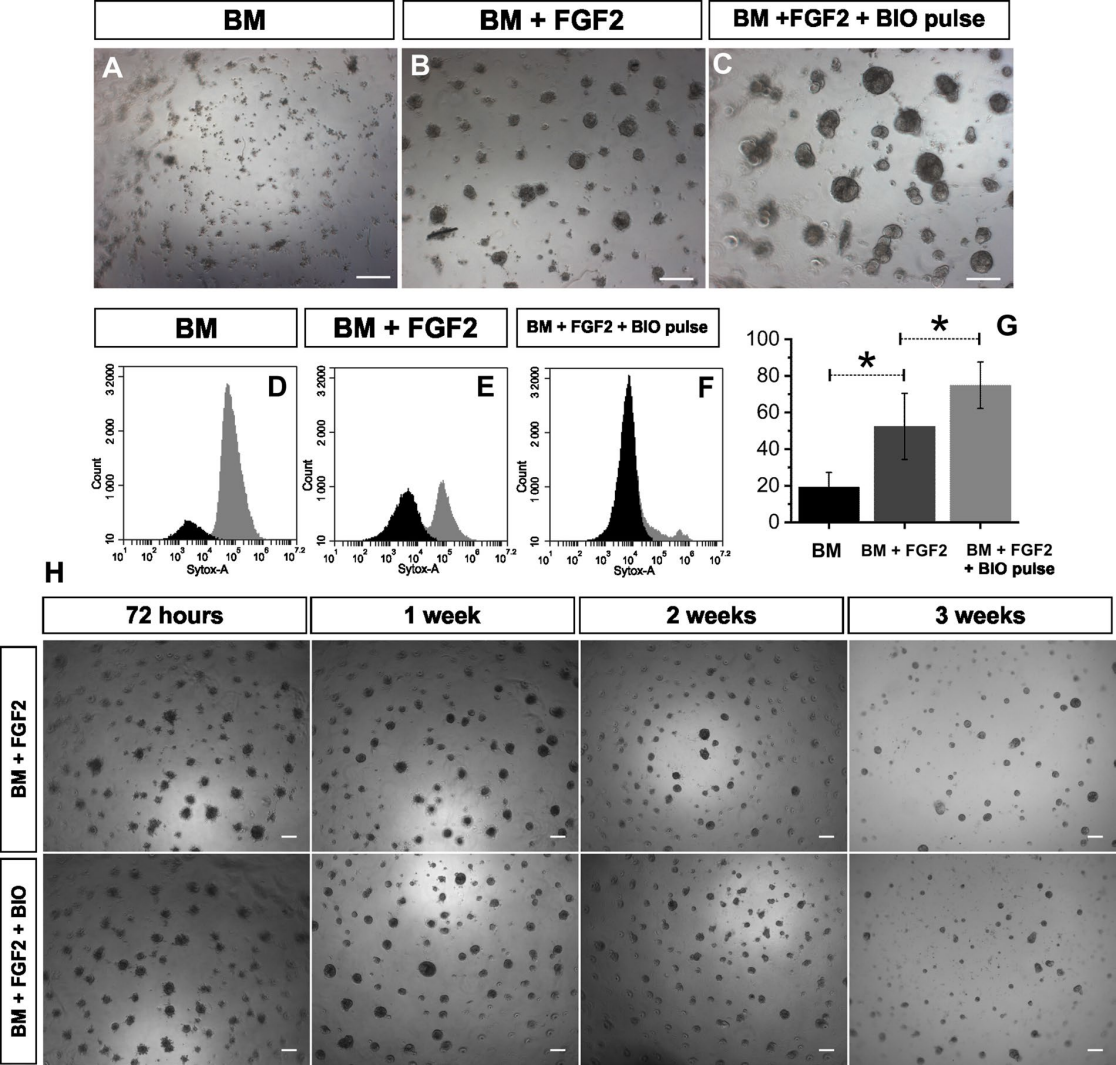
Self-organization, survival and maintenance of dissociated kidney mesenchyme in 3D culture. (**A**–**C**) Representative bright field images of dissociated KM after 72 hours of culture (n = 4). (**A**) Dissociated KM spheres cultured in basic media (BM) do not form spheres and decay in three days (**B**) Dissociated KM cultured in BM + FGF2 self-organizes into spheres. (**C**) Dissociated KM cultured in BM + FGF2 with pulse of 10 µM BIO 1 hour. FGF2 promotes dissociated KM cells to form aggregates resembling pretubular aggregates of an intact kidney. Further culture facilitates aggregates to form spheres and enhances dissociated KM maintenance. Dissociated KM cultured in FGF2 + 10 µM BIO 1 hour pulse self-organized to large, dense spheres. (**D**–**F**) Flow cytometric graphs of cell survival analyzed by cytotoxicity Sytox assay after 72 hours of culture. (**D**) Dead cell fraction (gray) dominates in dissociated KM cultured with BM. (**E**) Viable cell fraction increases drastically in dissociated KM grown with addition of FGF2. (**F**) BM + FGF2 + 10 µM BIO 1 hour pulse additionally boosts cell survival in dissociated KM spheres. (**G**) Quantification of flow cytometry data. Sytox assay’s values are inverted to present viability in Y axis. Dissociated KM survival increase from 20.1% ± 8.8% in BM culture to 57.4% ± 20.9% when cultured in the presence of FGF2, and reached 79.8% ± 14.4% in dissociated KM cultured in BM + FGF2 + BIO pulse. Asterisks indicate statistically significant difference (p < 0.05, paired sample t-test, n = 4, error bars - standard deviation). (**H**) Representative bright field images of dissociated KM cultures in BM + FGF2 (upper panel) and in BM + FGF2 + 50 nM constant BIO concentration. Dissociated KM cells form spheres and survive up to 3 weeks without major morphological differences between these two conditions. Scale bars in bright field images = 100 µm.

To characterize NPs self-organization *in vitro*, KM from E11.5 mouse embryos were microsurgically dissected from the UB and dissociated into single cells (Fig. 1A–C). Previous studies have shown that FGF family proteins are essential for the KM maintenance^27–29^. Therefore, basic culture medium (BM) was supplemented with 50 ng/ml of FGF2. We then plated 30,000 dissociated KM cells on top of 3D Matrigel per well of 96-plate in 200 µl either BM or BM + FGF2 (Fig. 1D, E respectively). Time-lapse imaging revealed that single cell suspensions self-organized into spheroids within 17 hours of culture (Supplemental video 1). To reassess the role of WNT in the self-organization and propagation of the NPs, the dissociated KM cell culture was pulsed with 10 µM of BIO for 1 hour (BIO pulse), thereafter the medium was replaced with fresh BM + FGF2 without BIO (Fig. 1F, Supplemental video 2). Bright field imaging after 72 hours of culture showed that dissociated KM cultured in BM-only did not form spheres and decayed with time (Fig. 2A), whereas in the BM + FGF2 and BM + FGF2 + BIO conditions dissociated KM self-organized into cell clusters comparably (Fig. 2B,C).

**Figure 3:**
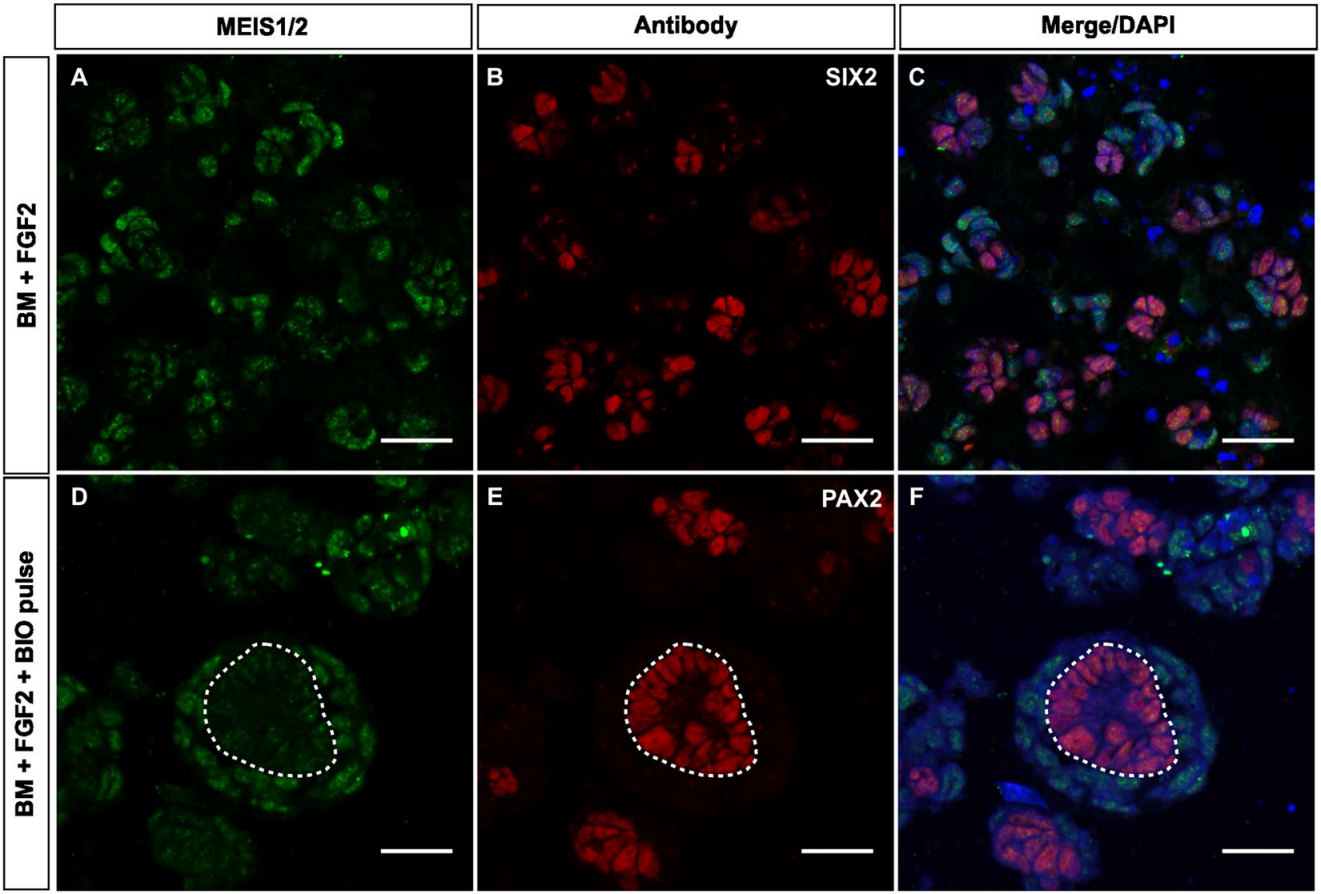
Dissociated kidney mesenchyme self-organizes into nephron progenitor-stromal niche, with the capacity to form pretubular aggregates. Representative immunofluorescence image of dissociated KM cultured for 72 hours in BM + FGF2 (**A**–**C**) and BM + FGF2 + BIO pulse (**D**–**F**). (**A**) Stromal lineage marker MEIS1/2 strongly stains most of the KM spheres cells. (**B**) Immunostaining for nephron progenitor marker SIX2 shows abundant NPs in KM spheres. (**C**) Merged image of separate channels for MEIS1/2 and SIX2 co-immunostaining, counterstained with nuclear stain DAPI shows spontaneous self-organization into spheres of both stroma and NPs resembling the niche of intact kidney. (**D**) MEIS1/2 immunostaining of BM + FGF2 + BIO pulse cultured spheres labels stromal cells (**E**) PAX2^+^ stains pretubular aggregate-resembling cell clusters. (**F**) Merged image of separate channels for MEIS1/2 and PAX2 co-immunostaining, counterstained with nuclear stain DAPI reveals that PAX2^+^ pretubular aggregates are wrapped inside of MEIS1/2^+^ stromal cells. Scale bars = 50 µm, F scale bar = 10 µm. Dotted line (**D**–**F**) marks pretubular aggregate border with stroma cells, n = 3.

To better understand the composition and self-organization of KM spheres we examined the cell identities by immunofluorescence at 72 hours of culture and compared these to E11.5 whole kidney reaggregation cultured in classical Trowell-type culture^12^. Cross-sections of the KM spheres were immunostained with different cell type markers to identify whether both stromal and nephrogenic lineages are present. This revealed that the spheres were composed of MEIS1/2^+^ stroma, SIX2^+^ and PAX2^ +^ NPs progenitors (Fig. 3A–F). The BM + FGF2 cultured KM spheres comprised of MEIS1/2 labeled cells (Fig. 3A, C) neighboring SIX2^+^ progenitors (Fig. 3B,C), whereas in spheres cultured in BM + FGF2 + BIO pulse PAX2^+^ NPs formed pretubular aggregate-like condensates, which were surrounded by MEIS1/2 positive stroma cells. Quantification of immunofluorescence images demonstrated, that KM spheres cultured in BM + FGF2 for 72 hours contained 48.56% ± 6.34% of SIX2^ +^ cells, whereas BIO pulse significantly reduced the SIX2^ +^ progenitors (4.31% ± 2.13%, p < 0.05, n = 6, Supplemental Fig. 1). Analysis of niche self-organization in the whole kidney dissociation and reaggregation culture revealed that E11.5 reaggregated kidneys were decreased in size at 72 hours (Supplemental Fig. 2) and were negative for NP marker SIX2 and nephron epithelial markers CDH1 and lotus tetragonolobus lectin (LTL) (Supplemental Fig. 3E–H). These immunostainings revealed that in our dissociated KM culture method, stroma and NPs spontaneously self-organize into stroma-NP niche with a capacity to recapitulate structures such as pretubular aggregates.

### FGF2 promotes maintenance of kidney mesenchyme spheres while BIO induces their expansion

To assess the survival of KM spheres in different conditions we re-dissociated and incubated the spheres with Sytox, a live cell impermeant agent allowing analysis of live cell proportions by flow cytometry (Fig. 2D–G). This revealed, in accordance with previous findings, that dissociated KM cultured in BM + FGF2 showed almost three times higher cell survival than those cultured in BM only (57.4% ± 20.9% and 20.1% ± 8.8% respectively, n = 4, Fig. 2D, E). A four-fold increase in cell survival was observed when dissociated KM cells were cultured with BM + FGF2 + BIO pulse (79.8% ± 14.4%, n = 4, Fig. 2B).

We then characterized proliferation in KM spheres for up to three weeks by EdU incorporation assay and Click-it fluorescent labeling of cells in S-phase of cell cycle (Supplemental Fig. 4). At 48 hours of culture, proliferating cell ratio of the KM spheres cultured in BM + FGF2 was 0.20 ± 0.05 while the same ratio was 0.38 ± 0.06 in spheres supplemented with BIO pulse (Supplemental Fig. 4A, B). This difference in proliferating cell ratios was abolished after one week of culture and remained similar also at three weeks of culture (Supplemental Fig. 4B). The deceleration of proliferation in KM spheres cultured with BIO pulse coincided with a rapid switch from progenitor to differentiation state in response to strong BIO pulse, as evident from immunofluorescence staining of one week cultured KM spheres (Supplemental Fig. 5).

### Kidney mesenchyme spheres WNT-independently undergo tubulogenesis

Several studies have reported that constant basic WNT activity, induced by continual presence of low dose of BIO or CHIR (another potent WNT-activator) facilitates NPs maintenance^17,30^. Since strong BIO pulse failed to maintain NP state and proliferation in long-term, we set to investigate the effect of low, constant BIO-dose on our 3D dissociated KM cultures. For this, the BIO concentration was titrated to identify appropriate concentration. After one week of culture, 50 nM BIO concentration maintained SIX2^+^ progenitors without much of the positivity for HPA (Supplemental Fig. 6) indicating maintenance of the progenitor status. No differences were observed by bright field microscopy between dissociated KM cultured in BM + FGF2 and BM + FGF2 + constant BIO in self-organization or KM sphere size (see Fig. 2H, one week time point). This concentration of BIO was chosen for following studies addressing progenitor status (SIX2^+^) and tubulogenesis as a readout of differentiation (lectin *Helix pomatia* agglutinin (HPA), a basolateral marker of nephron epithelium).

**Figure 4:**
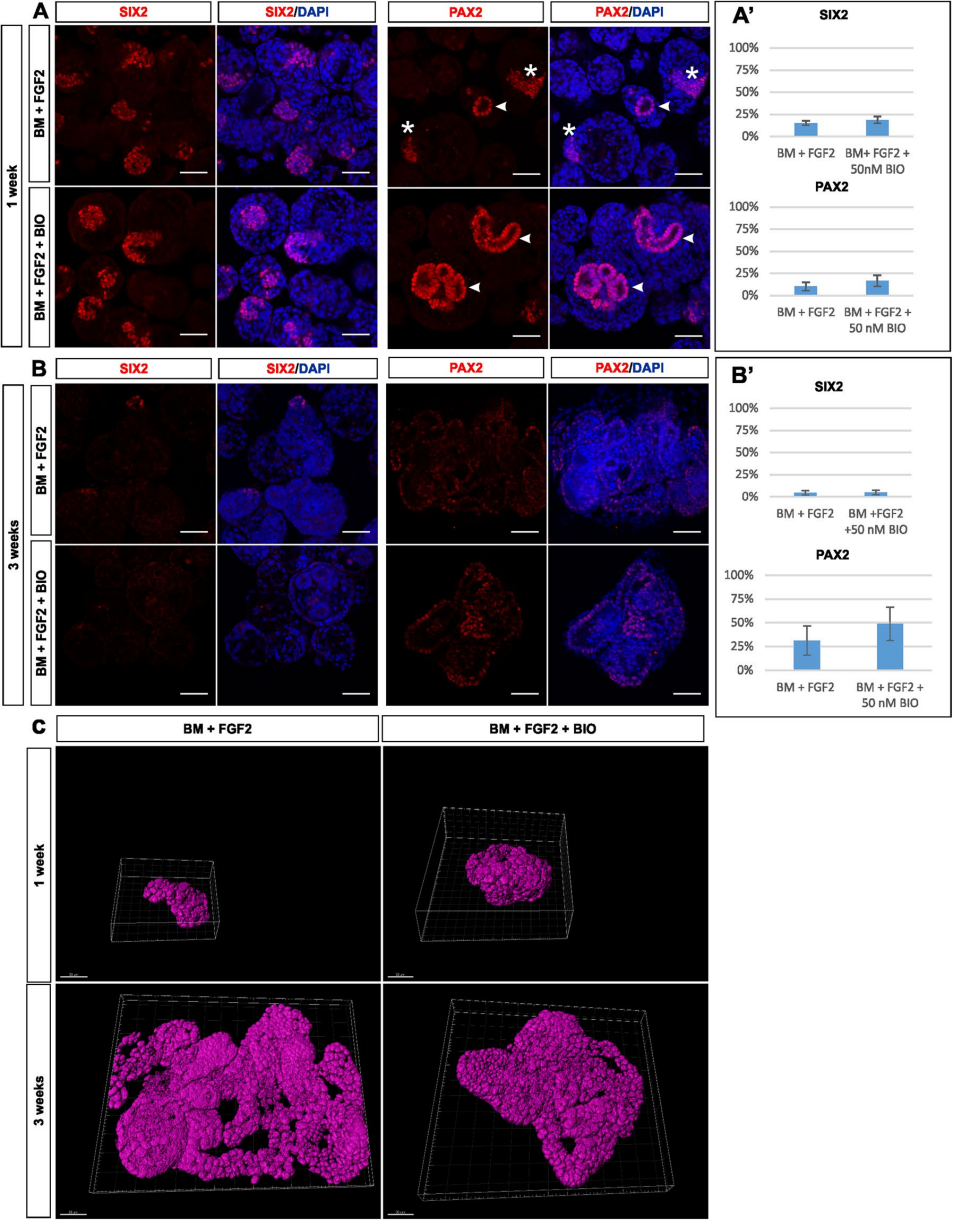
Nephron progenitors cultured without WNT activation spontaneously differentiate into tubules. (**A**) At one week, abundant SIX2^+^ NPs self-organize similarly into clusters regardless whether cultured in BM + FGF2 or BM + FGF2 + constant BIO (left panel). PAX2, a marker for progenitors and early nephron precursors, labels clustered cells (asterisks) and lumen forming tubular cells (arrowheads) in dissociated KM spheres cultured in BM + FGF2, whereas PAX2 labeling predominates in lumen forming tubular cells (arrowheads) when dissociated KM is cultured in BM + FGF2 + constant BIO (right panel). (**A**’) Quantification of staining at one week of KM cultured in BM + FGF2 or BM + FGF2 + constant BIO shows similar amounts of SIX2^+^ and PAX2^+^ progenitors (n = 3, p = 0.26). (**B**) At three weeks of culture, most of SIX2^+^ progenitor cells (left panel) have differentiated into PAX2^+^ complex tubular structures (right panel) in both conditions. (**B**’) Quantification of staining at three weeks of KM cultured in BM + FGF2 or BM + FGF2 + constant BIO shows small amount of SIX2^+^ progenitors 4.80% ± 3.13% and 4.46% ± 3.12% (n = 3, p = 0.93), respectively. In contrast, PAX2^+^ progenitors occupied 31.25% ± 15.33% and 48,73% ± 17,50% (n = 4, p < 0.05), of KM spheres respectively. **C**) 3D reconstruction rendered from PAX2 immunofluorescent stacks. Upper panel shows folded epithelial aggregates in BM + FGF2 and BM + FGF2 + constant BIO cultured KM spheres at one week time point. Lower panel shows complexed tubular network in both BM + FGF2 and BM + FGF2 + constant BIO KM spheres at three weeks of culture. Scale bar: A and B = 50 µm, C = 30 µm, perspective angle in C = 45°, representative images of 3 independent experiments.

We next analyzed the long-term maintenance of SIX2^+^ NPs in KM spheres cultured in BM + FGF2 and BM + FGF2 + constant BIO. Whole mount immunofluorescence staining showed that at one week of culture, SIX2^+^ NPs were maintained in both BM + FGF2 and BM + FGF2 + constant BIO cultured KM spheres (Fig. 4A, left panel). Quantification of the whole mount immunofluorescence images, using custom image analysis pipelines on the modular workflow system ANIMA^31^, showed no significant difference in ratios of SIX2^+^ and Pax2^+^-to-total cell counts between BM + FGF2 and BM + FGF2 + constant BIO (SIX2^+^ 15.32% ± 2.52%, 18.70% ± 3.78% n = 3, p = 0.25, and PAX2^+^ 10.32% ± 4.81%, 16.68% ± 6.28%, n = 3, p = 0.26, respectively, Fig. 4A’). Importantly, these data demonstrate a significantly longer maintenance of cultured NP cells than previously reported in such a simple culture system^22^. To assess the nephrogenic potential of NP cells cultured for one week in BM + FGF2 and BM + FGF2 + constant BIO we next exposed them to WNT activation, which revealed equal epithelization capacity comparable to that seen with naïve, freshly isolated MM (Supplemental Fig. 8). To test the long-term survival of NP cells in KM spheres we next analyzed SIX2 expression in spheres cultured for three weeks. Very few clusters of SIX2^+^ NP cells were detected in spheres cultured for three weeks regardless whether they were supplemented with FGF2 alone or together with constant BIO (4.46% ± 2.56% and 4.80% ± 2.56%, respectively, n = 3, p = 0.93, Fig. 4B, B’, and Supplemental Fig. 10). However PAX2^ +^ -to-total cell counts between BM + FGF2 and BM + FGF2 + constant BIO were much higher and with later culture condition causing significantly higher PAX2 expression (31.25 ± 15.33%, and 48.73% ± 17.50% respectively, n = 4, p < 0.05). At this stage, the sphere cultures appeared also not suitable for recombination experiments, which failed due to poor quality of the cells. Of note, epithelial nephron tubules were detected in KM spheres cultured in both conditions for three weeks indicating loss of NP cells by spontaneous differentiation (Supplementary Figs 9, 10).

We finally characterized nephrogenesis in the KM spheres cultured for one and three weeks. Staining with PAX2, a protein upregulated in nephron precursors undergoing epithelialization^32^, revealed PAX2^+^ cell aggregates, reminiscent of pretubular aggregates and lumen containing nephron tubules in spheres cultured with BM + FGF2 for one week (Fig. 4A, right panel). In BM + FGF2 + constant BIO conditions PAX2^+^ cells were predominantly found in lumen forming tubules (Fig. 4A, right panel arrowheads). This suggests that the MET was initiated, and to some extent also completed at one week of KM spheres culture. At three weeks, however, we observed a robust PAX2 signal with varying intensities in KM spheres cultured in BM + FGF2 (Fig. 4B, left panel). To ascertain the presence of the tubules, we performed 3D reconstruction of PAX2 immunofluorescence within KM spheres cultured for one and three weeks. At one week of culture, we again confirmed the presence of PAX2^+^ in the early cell aggregate of spheres cultured in BM + FGF2 and larger aggregates in spheres cultured in BM + FGF2 + constant BIO (Fig. 4C, upper panel). At three weeks a network of PAX2 labelled complex tubular structures were observed in both BM + FGF2 and BM + FGF2 + constant BIO cultured KM spheres (Fig. 4C). We have further confirmed that these tubules are CDH1 positive (Supplemental Fig. 10), however lack of segment specific markers such as LTL in CHD1 + tubules suggests that the nephron epithelium is fairly immature. Taken together, the panel of immunofluorescence staining and their 3D reconstructions indicate that KM spheres spontaneously differentiate into tubular epithelium even without activation of WNT signaling.

## Discussion

The organization of an *in vivo* NP niche involves presence of all three tissue layers contributing to normal kidney organogenesis, namely KM itself, stroma and UB epithelium. *In vitro*, the self-organization and propagation of kidney nephron progenitors require activation of a complex mixture of different cascades aiming to mimic signals normally secreted by UB and stroma^17,30^. Simple systems, which allow manipulation of the cells, have utilized dissociation and re-aggregation together with the UB-derived inductive signals such as GSK3 inhibition, UB itself or spinal cord expressing several *Wnt*s^1,12,20,22,33^. Until recently, *in vitro* expansion of nephron progenitors was not possible and current protocols now in use are not only expensive but also tedious^17,34^.

Various *in vivo* and *in vitro* studies have indicated that FGF signaling is indispensable for survival and maintenance of metanephric mesenchyme^18,22,27–29^. Our 3D cell culture model confirms this. Contrary to previous studies, here we present a simple 3D culture method to aggregate and propagate dissociated KM into spheres resembling NP niche and maintaining nephrogenic potential up to one week. This new method utilizes the 3D Matrigel in combination with FGF2 and PP2 to generate conditions allowing self-organization into nephron progenitors and stromal cells where both lineages interact with each other and support their maintenance. As indicated by previous studies, a clear lineage separation of the nephron and stromal progenitor is established early in renal differentiation^35–37^. Stromal compartment in cooperation with ureteric epithelium is also shown to be crucial for NP self-renewal and differentiation^38–40^. In the light of a growing interest towards generating kidney organoids from human cells^41,42^, our culture method presents more simple approach by providing an opportunity to investigate the cell aggregation^43^, self-organization, cross-talk between stroma and NPs in the conditions where signals from ureteric epithelium or other inductive tissues are not needed.

Likely, the most interesting finding with our newly developed dissociated KM culture is the observation that NPs within KM spheroids, cultured without exogenous WNT-activation, undergo spontaneous epithelization. This exciting finding underlines the robustness of the nephron induction and tubulogenesis programs, implying that similarly to rat kidneys^44^, signaling pathways other than just GSK3β/WNT-activation supplement regulation of self-renewal and differentiation decisions in mouse KM. Indeed, Schmidt-Ott and colleagues previously identified conserved UB tip expressed gene signature in mouse and rat kidneys, and demonstrated that e.g. secreted cytokine receptor, cytokine receptor-like factor 1 (CLF-1) in complex with its ligand induces tubulogenesis in isolated mouse MM^45^. Moreover, several studies also support the involvement of FGF induced signaling in differentiation of nephrons^43,46,47^. Thus, the robust and easy 3D culture method presented here provides a tool to study niche-NPs interaction and can be utilized to generate new knowledge about NPs characteristics and nephron differentiation.

## Material and Methods

### Tissue culture

Mouse embryonic kidneys were microdissected from E11.5 NMRI embryos as described^48^. The kidneys were dissected in PBS, and subsequently treated with collagenase type IV for 15 min at 37 °C. Thereafter, the kidney mesenchyme (KM) was separated from the ureteric epithelium (UB) by microdissection in the DMEM supplemented with 10% fetal calf serum. The KM was incubated with TrypLE Express and dispersed into single cells in Basic Media (BM) (DMEM/F12, 10% fetal calf serum (FCS), and 10 µM PP2 (to reduce neuronal outgrowths and augment sphere aggregation) or BM with 50 ng/ml FGF2^9^. The cell suspension was seeded on normal growth factor 3D Matrigel (Basement Membrane Matrix from EHS mouse tumor, BD Biosciences), at the density of 3 × 10^4^ cells/well, 50 µl/well to Lab-Tek Chamber Slide system 16 chamber glass slide (LabTek/ThermoFisher Scientific) or to a flat bottom 96 well plate (Costar/Corning Inc.). For BIO ((2′Z,3′E)-6-Bromoindirubin-3′-oxime; Calbiochem) pulse experiments, BIO was added at a final concentration of 10 µM during cell plating and removed after 1 hour by replacing medium with 200 µl of fresh BM + FGF2 without BIO. For low dose experiments, BIO was added to 50 nM final concentration and kept constant during the culture. All media were replaced with fresh after three days.

### Embryonic kidney dissociation and reaggregation

Embryonic kidneys were dissociated and reaggregated as described in (Unbekandt and Davies, 2010)^12^ with following modifications: E11.5 embryonic kidneys were isolated as described^48^, the isolated kidneys were dissociated into single cells by pipetting after incubation of 5 min in TrypLE at 37 °C. The digestion was blocked by washing in 10 parts of DMEM supplemented in 10% FCS. Cell were counted and at least 80,000 cells were used for reaggregation by centrifugation at 500 × g for 3 min. Pellets were transferred on nucleopore carbon filter and cultured on Trowell-type system at air liquid interface in DMEM supplemented with 10% FCS.

### Tissue recombination

After five days of culture, the KM spheres were recovered from Matrigel and transferred on top of a piece of E11.5 mouse spinal cord (Supplementary Fig. 7). The recombinant tissue was cultured in Trowell type tissue culture for 72 hours, followed by fixation with 4% PFA for 1 hour at room temperature and subjected to immunofluorescence staining.

### Immunofluorescence

The KM spheres were fixed for 20 min at RT with 4% PFA (unless otherwise stated), washed with PBS, pelleted in 1.5 ml low protein binding tubes, embedded in Richard-Allan Scientific HistoGel (for paraffin sections) or OCT embedding compound (for cryosections), and subsequently cut at 5 µm section in microtome or cryostat. The sections were blocked with secondary antibody host specific 10% serum for 1 hour at RT. Samples were incubated with primary antibodies overnight at +4 °C, followed washes in PBS and 1 hour incubation with secondary antibodies. The samples were then counterstained with 10 µg/ml of Hoechst or DAPI, and mounted for imaging.

### Whole mount immunofluorescence

The KM spheres were fixed for 30 min at RT with 4% PFA, washed with PBS, pelleted in 1.5 ml low protein binding tubes and pellets were transferred on microscopic slide. The pellets were permeabilized for 20 min with PBS Triton x-100 0.5%, blocked for 1 hour with secondary antibody host specific 10% serum at RT. Samples were rinsed with PBS and incubated with primary antibodies at +4 °C o.n., followed by wash and incubation with secondary antibody o.n. at +4 °C. After wash samples were counterstained with DAPI mounted and imaged.

### Proliferation assay

At the indicated time points the proliferating cells were labeled with 10 µM of 5-ethynyl-2′-deoxyuridine (EdU) for 2 hours and detected by Click-IT EdU Imaging Kit (AlexaFluor-594 conjugated, Life Technologies) according to the standard protocol. Subsequently, the samples were co-immunostained as described above.

### Flow cytometric analysis of cell viability

After 72 hours in culture the KM spheres were harvested from Matrigel, transferred to low protein binding tubes, and collected by centrifugation and subsequently dissociated into single cells by TrypLE Express. Cells were stained with Sytox Red Dead Cell Stain (ThermoScientific) according manufacturer’s instruction, and analyzed with Accuri Flow Cytometer (BD Biosciences) at the HiLIFE Biomedicum Flow Cytometry core facility.

### Imaging and image analysis

The samples were imaged by Zeiss AxioImager Z1 or Leica SP8 confocal microscope. Bright field images were taken by Nikon Eclipse Ti-E. The proliferation and protein expression ratios (labeled cells to-total cell count) were quantified using custom developed pipelines on the modular workflow system ANIMA^31^. In short, images were pre-processed, segmented for cell nuclei by Graphcut algorithm using Farsight library^49^, and mapped for mask, which was used to measure intensity of label channel. The intensity values and their standard deviations were clustered by k-mean algorithm to determine negative staining threshold and signal intensity classes. KM spheres 3D structure was reconstructed using Imaris software (Bitplane) from convoluted (Huygens Professional software, SVI) immunofluorescence and DAPI counterstained-stained confocal microscopy images. Imaging, image deconvolution and 3D rendering has been performed at HiLIFE Biomedicum imaging core facility.

### Data analysis and statistical methods

For all figures the independent experiments (different mouse litters with no less than 8 embryos) were performed at least three times. The proliferation ratio was calculated from at least three independent experiments, each of which contained pooled KM sample from at least eight embryos. To determine the proliferation ratio and cell survival percentage no less than 10,000 cell counts were used. For SIX2 and PAX2-to-total cell count positive cell quantification, images form at least 3 independent experiments were used and calculated as described in image analysis section above. The independent two-sample t-test (significance p < 0.05) analysis was performed on the means of the ratios (calculated with OriginPro statistical analysis software).

### Ethical approval

All methods were carried out in accordance with relevant guidelines and regulations. All animal husbandry and experimental procedures were approved by EU legislation and Finnish Animal Care and Use Committee.

### Significance statement

The kidney mesenchyme (KM), also known as metanephric mesenchyme, contains both nephron progenitors (NPs) and stromal cells. *In vitro* maintenance and propagation of dissociated KM and uninduced NPs is not trivial, and current culture protocols rely on complex procedures. This study demonstrates that a combination of three dimensional (3D) cell culture and Fibroblast Growth Factor 2 (FGF2) expands NPs that self-organize into spheres resembling the niche. They also form epithelial tubes without inductive tissues or exogenous source for WNT activation. This robust and easy 3D culture method provides a tool to study niche-NPs interaction and can be utilized to generate new knowledge about NPs characteristics.

## Supplementary information

supplemental video 1

supplemental video 1

suppelmental information

## Data Availability

The datasets and quantification pipelines generated during and/or analyzed during the current study are available from the corresponding author on reasonable request.
