# Supplementary material for: Simple 3D culture of dissociated kidney mesenchyme mimics nephron progenitor niche and facilitates nephrogenesis Wnt-independently
Source: Sci Rep. 2019 Sep 17;9:13433. doi: 10.1038/s41598-019-49526-x (PMC6748995; doi:10.1038/s41598-019-49526-x)
Supplement: Supplementary file 3 — suppelmental information [file 41598_2019_49526_MOESM3_ESM.pdf]

Supplemental information

Simple 3D culture of dissociated kidney mesenchyme mimics nephron progenitor niche and facilitates nephrogenesis Wnt-independently

Arvydas Dapkunas, Ville H Rantanen, Yujuan Gui, Maciej Lalowski, Kirsi Sainio, Satu Kuure, Hannu Sariola

Antibodies and fluorescent reagents

Primary antibodies included rabbit polyclonal anti-Pax2 (Life Technologies, 1:200); rabbit polyclonal anti-Six2, (Proteintech, 1:200); rabbit polyclonal anti- Nephrin (Karl Tryggvason, 1:1000), rabbit polyclonal anti-Wt1 (Santa Cruz 1:500), goat polyclonal anti-Meis1/2 (Santa Cruz, 1:200), mouse anti-CDH1 (BD Transduction Laboratories 1:400).

Secondary antibodies and lectin conjugates were as follows: anti-rabbit AlexaFluor 488, anti-rabbit AlexaFluor 568, anti-mouse AlexaFluor 488, anti-mouse AlexaFluor 568, anti-goat AlexaFluor 488 (Thermo Scientific, 1:400); anti-mouse Cy-5, (Jackson Immunoresearch Laboratories, 1:200); Lectin HPA AlexaFluor 488 and AlexaFluor 647 (Life Technologies, 2.5 μg/ml); Fluorescein-Lotus Tetragonolobus Lectin, (Vector Laboratories, 20 μg/ml).

Supplemental figures

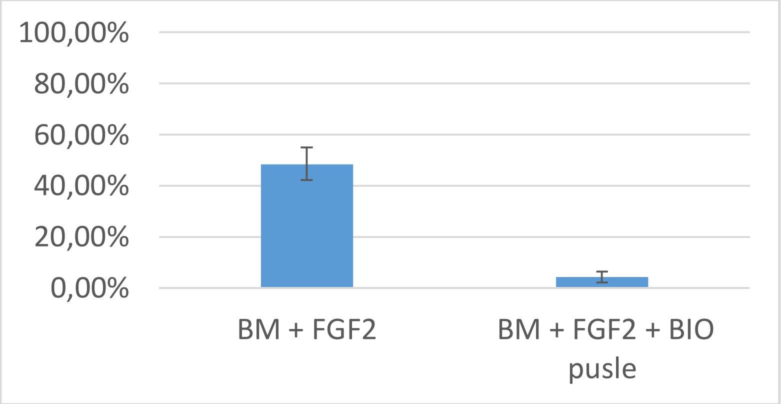

Supplemental Fig. 1. Strong BIO pulse reduces SIX2 progenitors after 72 hours in culture. Graph depicts quantification of SIX2 immunofluorescence images of KM spheres cultured in BM + FGF2 and BM + FGF2 + BIO pulse. KM spheres cultured in BM + FGF2 for 72 hours contained 48.56% ± 6.34 % $SIX2^+$ cells, whereas BIO pulse significantly reduced the $SIX2^+$ progenitors to 4.31% ± 2.13%, n=6, t-test $p<0.05$.

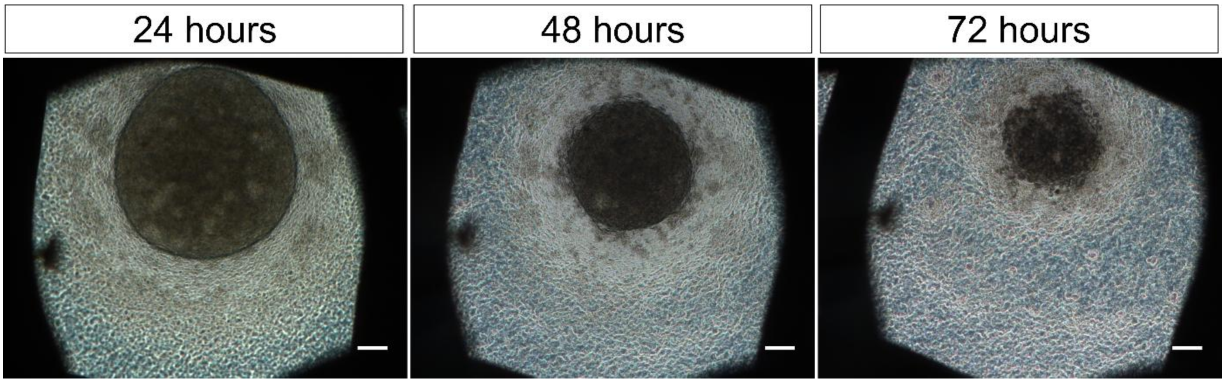

Supplemental Fig. 2. E11.5 kidney reaggregates cultured in classical Trowell-type system. Representative panel of phase contrast time-lapse images of E11.5 reaggregated kidneys cultured in DMEM medium. The aggregates reduce in size during time course of 72 hours. Scale bar = 100 µm, n=3.

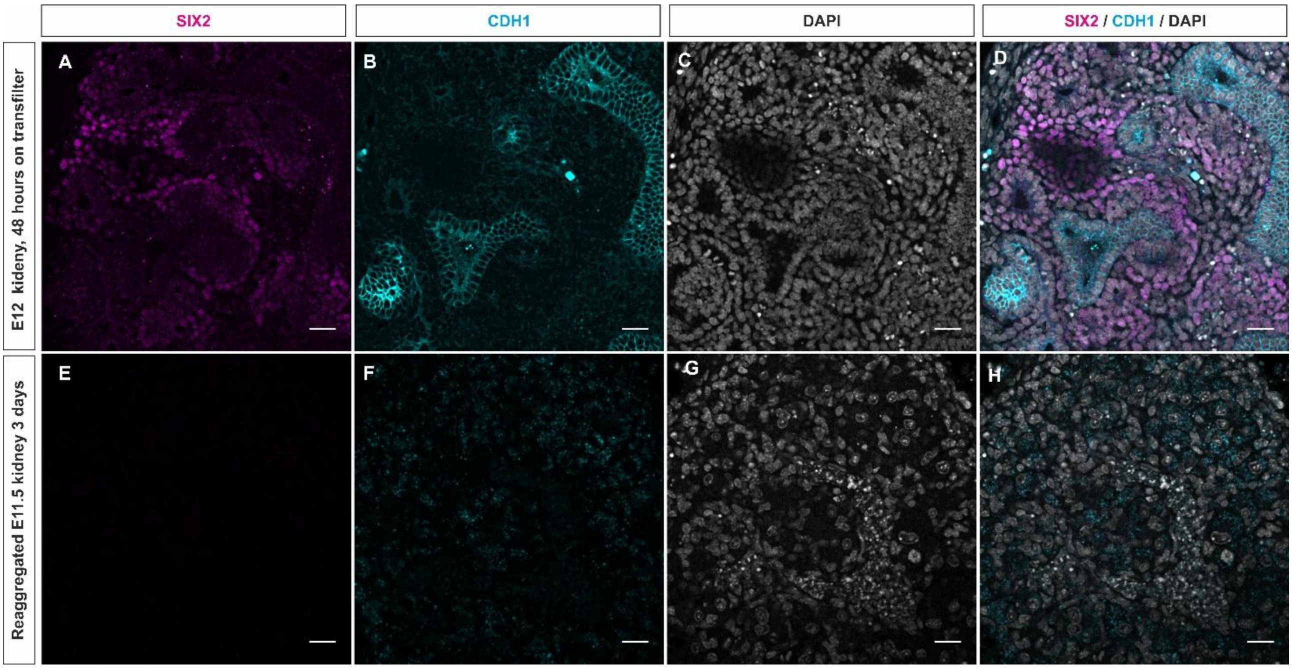

Supplemental Fig. 3. E11.5 kidney reaggregates fail to maintain nephron progenitors after 3 days in Trowell-type culture. A – D co-immunofluorescence staining of E12 kidney culture 2 days in Trowell-type culture and stained for NP's marker SIX2 (magenta) and epithelial marker CDH1 (cyan). The co-immunostaining shows normal embryonic kidney patterning with SIX2 positive cap mesenchyme in A and D, and CDH1 positive UB and nephron epithelium in B and D. Dissociated and reaggregated E11.5 kidneys fail to reproduce embryonic kidney niche and lose SIX2$^+$ progenitors (E) as well as degrade the epithelial structures (G). Scale bars = 50 µm, n=3.

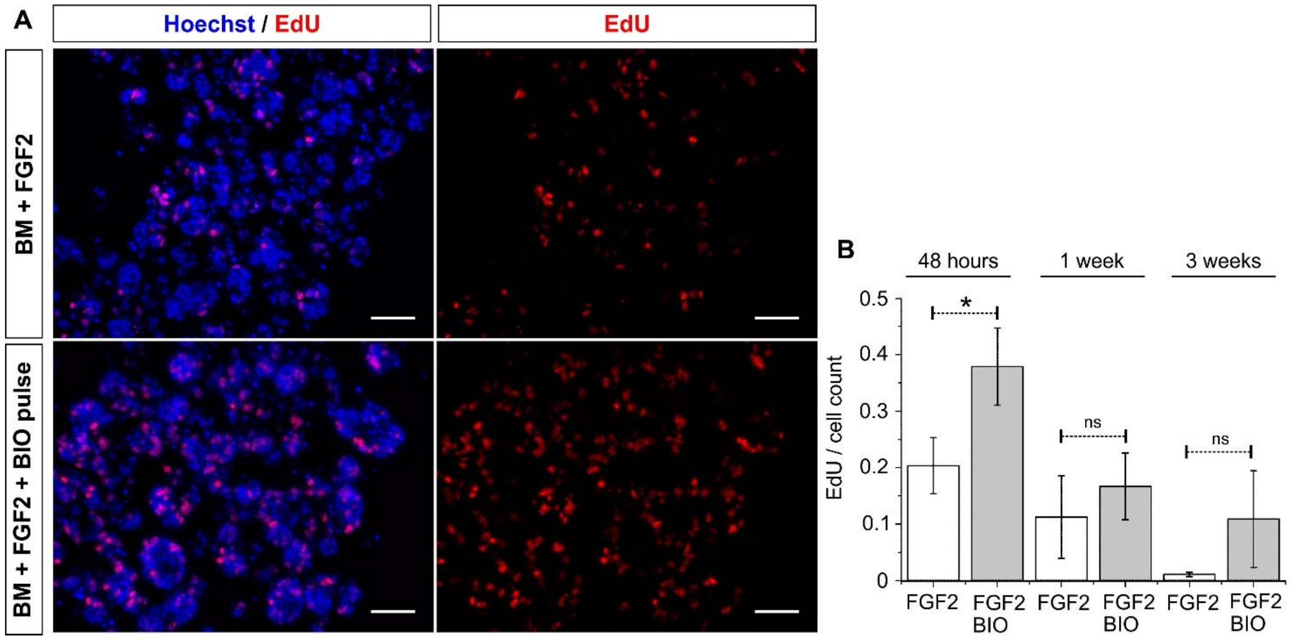

Supplemental Fig. 4. EdU incorporation assay reveals proliferation in KM spheres. A) A panel of representative immunofluorescence images after double staining of KM spheres with nuclear marker Hoechst (blue) and EdU (red) at 48 hours of culture. Scale bars = 50 µm, n=3. **B**) Plot showing quantitative results of the proliferation assay followed up for three weeks. KM spheres cultured in BM + FGF2 + 10 µM BIO 1 hour pulse showed two-fold statistically significant increase in proliferation rate at 48h, but failed to maintain proliferative advantage through one and three weeks of cultures. (* - $p<0.05$, ns - not significant, n=3, error bars – standard deviation).

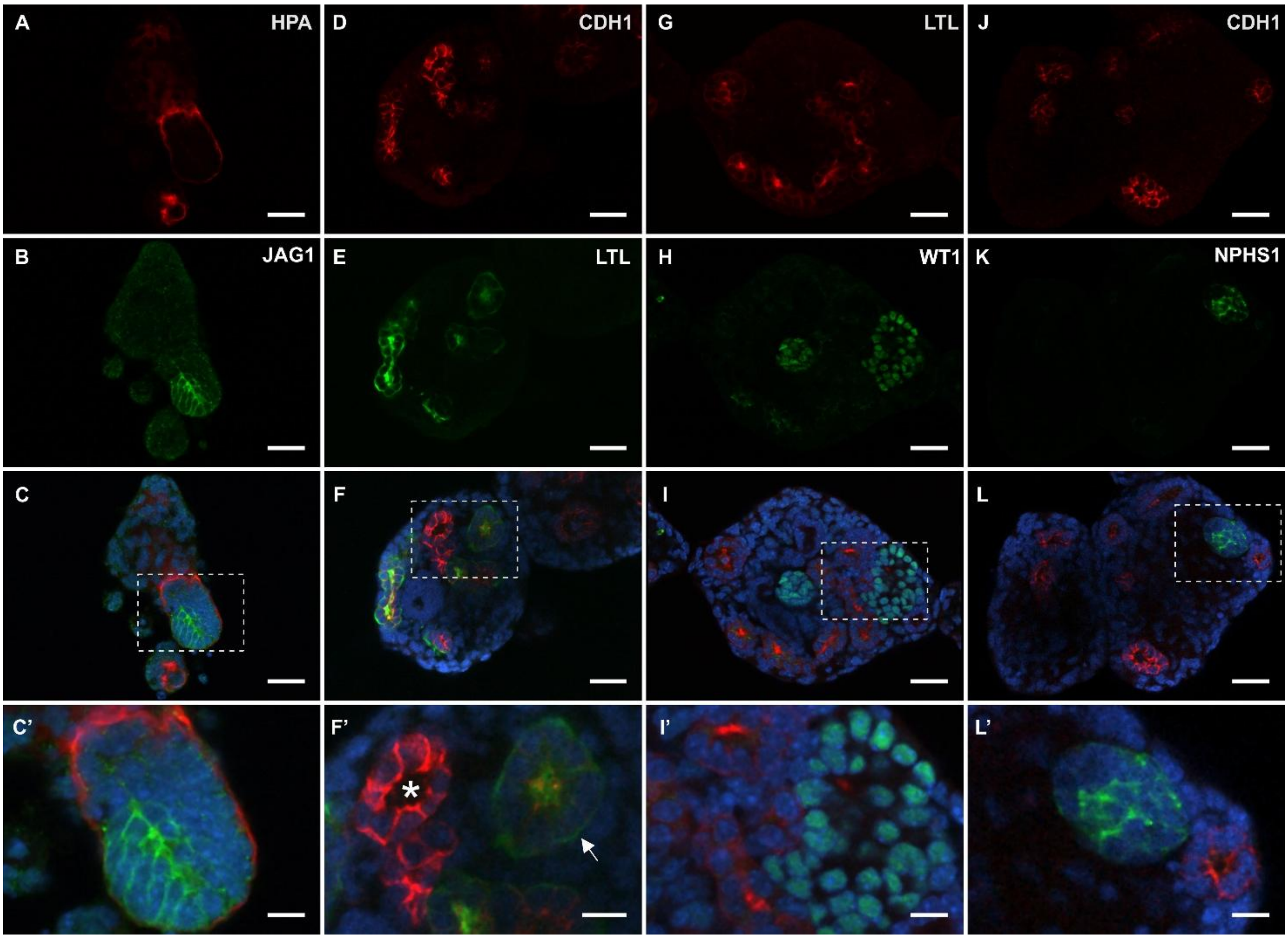

Supplemental Fig. 5. BIO pulse induces KM spheres to express molecular markers of nephron segments. Panel of representative whole mount confocal immunofluorescence images of KM spheres cultured in BM + FGF2 + 10 µM BIO 1 hour pulse and analyzed after one week. **A**) Lectin *Helix pomatia* agglutinin (HPA), a basolateral marker of kidney epithelium, wraps around epithelializing cells. **B**) KM spheres are positive for JAG1, which is a marker of proximal nephron segment. **C**) Merge of A and B images revealed unilateral polarization of JAG1 (green) signal within HPA (red) positive epithelial tubule suggesting a proximal distal patterning of the nephron tubule. **C'**) Magnification of the dashed area in C. **D**) KM-derived epithelial tubules are positive for E-CADHERIN (CDH1), an epithelial marker. **E**) Lotus lectin (LTL) staining displays typical proximal tubule pattern. **F**) Merge of D and E images shows $CDH1^{+}/LTL^{-}$ distal commitment. **F'**) Magnification of the dashed area in F, distal tubule depicted by an asterisk, proximal tubule by an arrow. **G**) LTL stained proximal tubules. **H**) Podocyte precursors stained with WT1. **I**) In the merged images, the proximal tubules are found adjacent to $WT1^{+}$ (green) podocyte precursor clusters. **I'**) Magnification of the dashed area in I. **J**) CDH1 immunostained epithelial tubules. **K**) Cell clusters labeled with podocyte lineage marker Nephrin (NPHS1). **L**) CDH1 (red) positive epithelial tubule localized next to NPHS1 (green) positive cell cluster. **L'**) Magnification of the dashed area in L. The staining showed that KM spheres supplemented with BM + FGF2 + BIO pulse and cultured additional week underwent MET and

expressed molecular markers of main nephron segments. Scale bars A-L 25 μm, C', F', I', L' 10 μm, n=3.

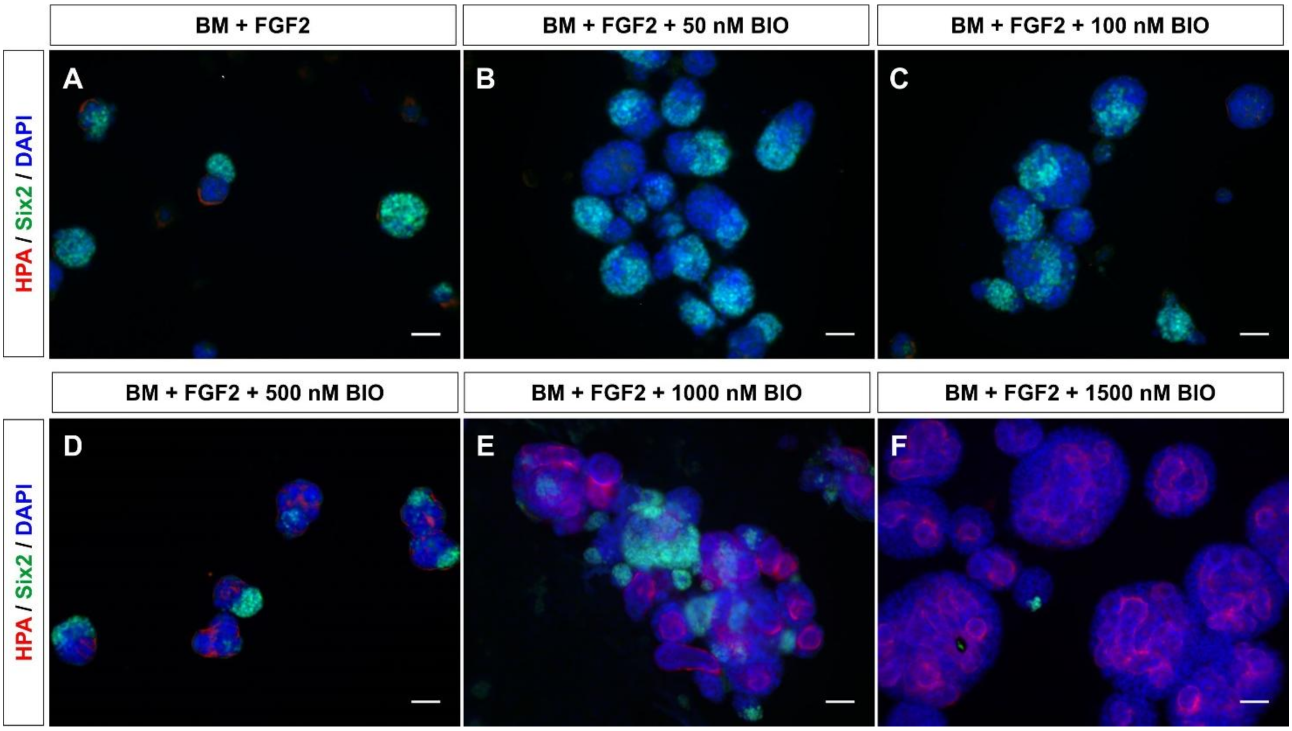

Supplemental Fig. 6. Dose dependent BIO effect on nephron progenitor fate in dissociated KM cultured for one week. A) Dissociated KM cultured in BM + FGF2 **B**) Dissociated KM cultured in BM + FGF2 + 50nM constant BIO **C**) Dissociated KM cultured in BM + FGF2 + 100nM constant BIO. **D)** Dissociated KM cultured in BM + FGF2 + 500nM constant BIO **E**) Dissociated KM cultured in BM + FGF2 + 1000nM constant BIO **F**) Dissociated KM cultured in BM + FGF2 + 1500nM constant BIO. **A-C** Spheres predominantly contain large clusters of SIX2$^+$ cells. **D-E** KM spheres show increasing amount of epithelization with increased, constant BIO dose as shown by increase in lectin HPA staining (red) with mutual reduction in SIX2$^+$ progenitors. Based on ratio of SIX2$^+$ progenitors to sphere size and lack of HPA staining, the 50 nM concentration of BIO was selected to test progenitor maintenance in long-term cultures. Scale bars = 25 μm, n=3.

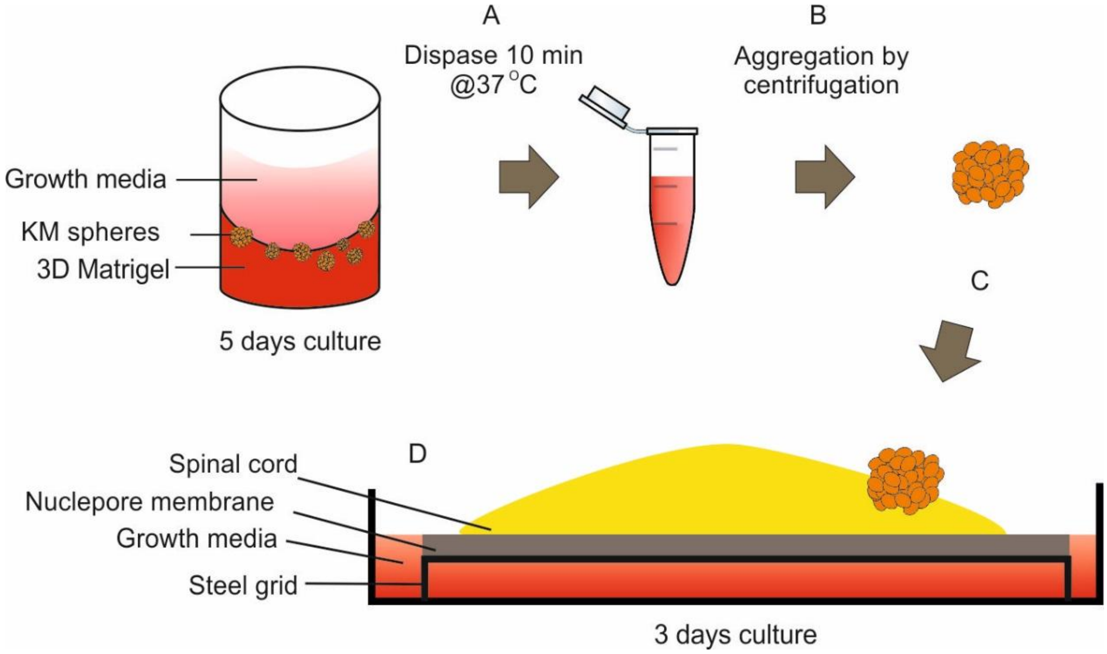

Supplemental Fig. 7. Experimental setup for recombination of the KM spheres with the heterologous inducer, the mouse embryonic spinal cord. A) 5 days after of culture, the KM spheres are recovered from 3D Matrigel by enzymatic digestion with Dispase. **B**) The free floating KM spheres were then transferred to microcentrifuge tubes and centrifuged to aggregate. **C**) The aggregated KM spheres pellets were then placed directly on top of the mouse embryonic spinal cord (yellow hill). **D)** The spinal cords (yellow hill) together with KM spheres were subsequently cultured in Trowel type tissue culture for three days.

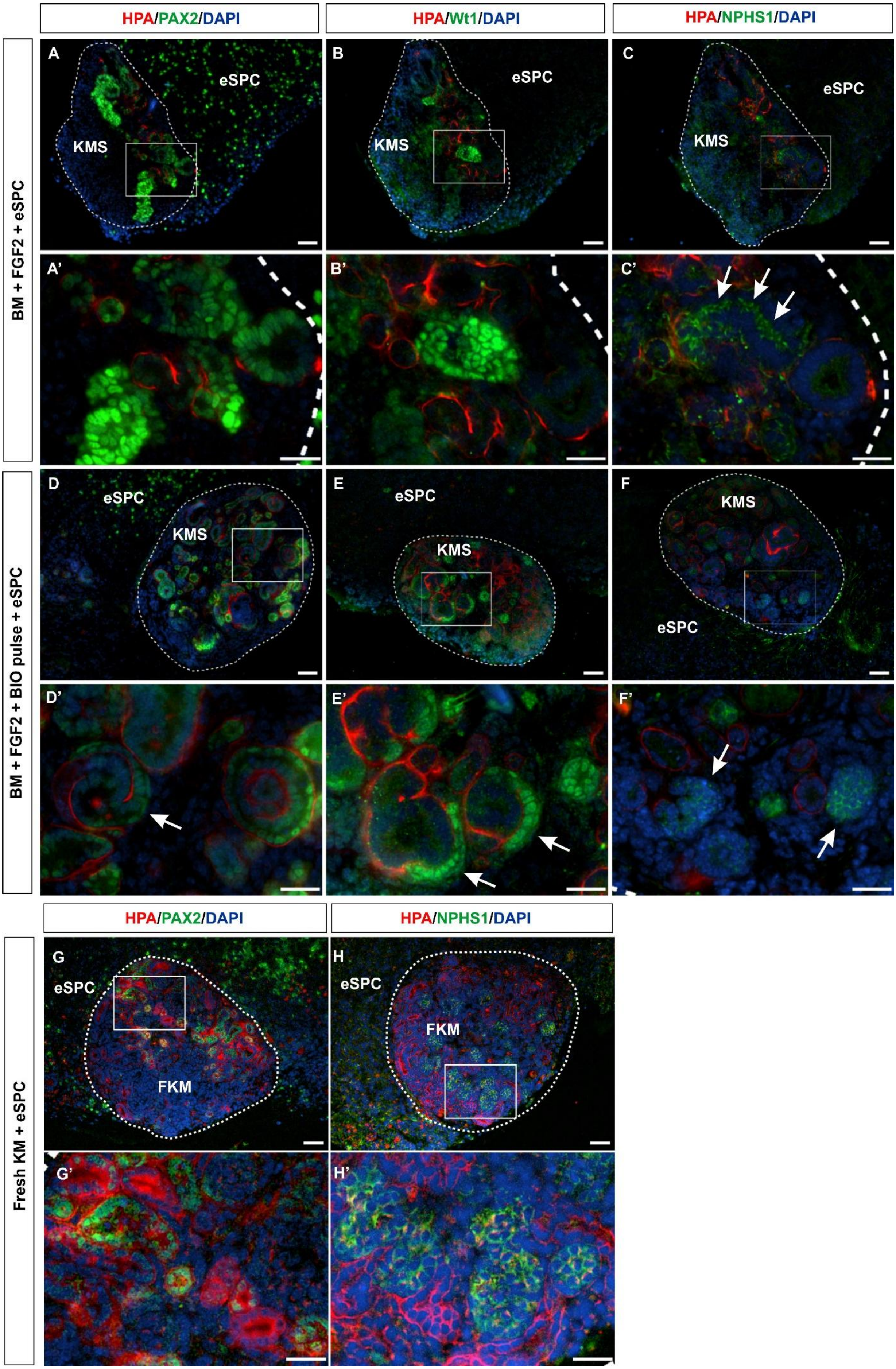
HPA/PAX2/DAPI
HPA/Wt1/DAPI
HPA/NPHS1/DAPI
BM + FGF2 + eSPC
A
B
C
eSPC
KMS
A'
B'
C'
BM + FGF2 + BIO pulse + eSPC
D
E
F
D'
E'
F'
HPA/PAX2/DAPI
HPA/NPHS1/DAPI
Fresh KM + eSPC
G
H
FKM
G'
H'

Supplemental Fig. 8. Nephron progenitors respond to WNT stimulation by epithelization and expression of podocyte precursor molecular markers. Panel of representative immunofluorescence images of KM spheres cultured for 5 days and then recombined with embryonic spinal cord (eSPC) for 72 hours. KM sphere pellets were placed on top of the spinal cord, and delineated in the figure by dashed lines. Basal lamina marker lectin HPA (red) indicates epithelial structures, while distinct antibody fluorescent-labeling are shown in green. Cell nuclei were counterstained with DAPI (blue). **A**) KM spheres cultured in BM+FGF2 and recombined with eSPC underwent nephrogenesis as seen by $PAX2^+$ and lectin HPA^+ tubules. **B**) Podocyte marker WT1 visualizes cell clusters around lectin HPA^+ tubules in KM spheres cultured with FGF2 and recombined with eSPC. **C**) Nephrin expression (NPHS1), demarcating podocytes of glomeruli, was observed adjacent to HPA positive tubules in KM spheres cultured with FGF2 and recombined with eSPC (**C'** arrows point to NPHS1 positive staining). **D**) Complex tubular structures positive for lectin HPA and PAX2 are noticeable in KM spheres cultured in BM +FGF2 + BIO pulse and recombined with eSPC. (**D'** Arrow points to S-shape body typical for developing nephron *in vivo*). **E)** KM spheres cultured in BM +FGF2 + BIO pulse stained for WT1 and Lectin HPA (**E'** arrows point to WT1 labeled maturing "half-moon" shaped glomeruli). **F**) The cells labeled with NPHS1 and lectin HPA (arrows point to NPHS1 positive cell clusters). **G)** Freshly isolated KM recombined with eSPC and immunostained for PAX2 and lectin HPA shows subset of HPA^+ tubules label with PAX2. **H)** In freshly isolated KM recombined with eSPC and immunostained for NPHS1 and lectin HPA, abundant glomerular-like aggregates are $NPHS1^+$ within the vast network of HPA^+ tubules. Scale bars = 50 µm, n=3. KMS – kidney mesenchyme spheres; eSPC – embryonic spinal cord; FKM – freshly isolated kidney mesenchyme. **A', B', C', D', E', F', G', and H'**) Magnification of a squared area in the full scale images.

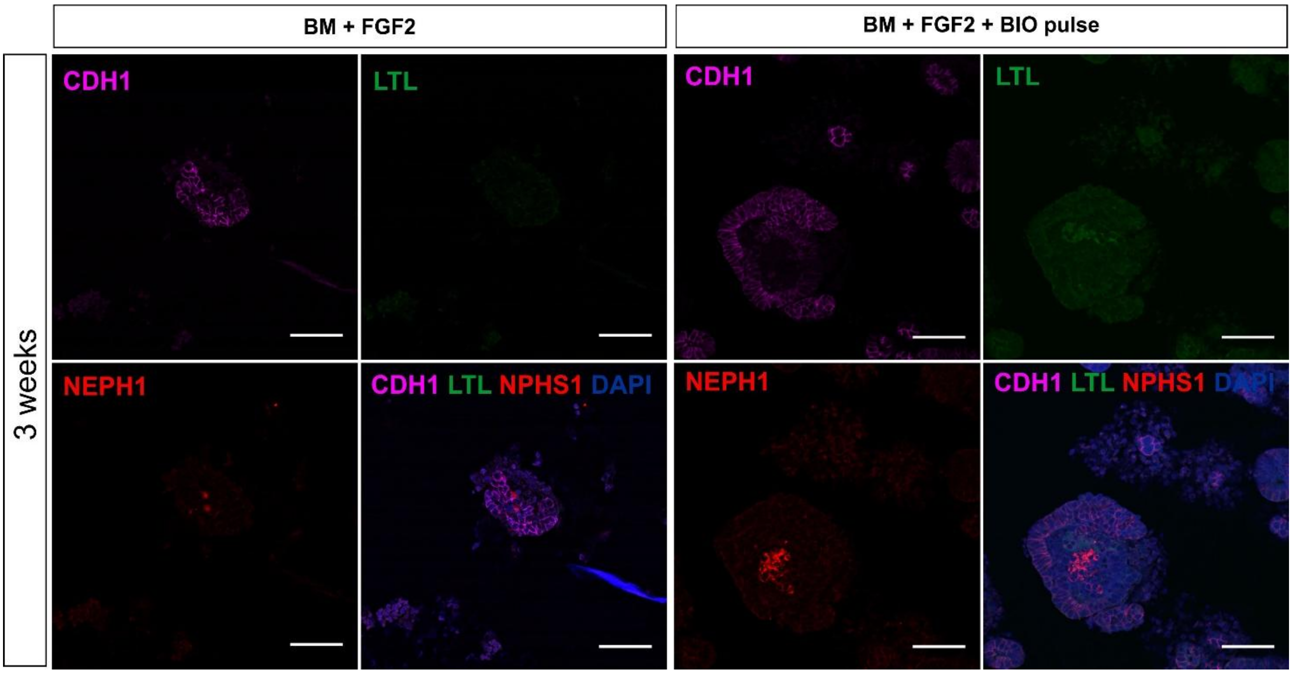

Supplemental Fig. 9. KM spheres differentiate into nephron tubules WNT independently. A panel of immunofluorescence images of samples cultured for three weeks in BM + FGF2 or BM + FGF2 + BIO pulse followed by staining of indicated nephron segment markers. Left side panel shows that KM spheres cultured in BM + FGF2 contain CDH1 positive tubules but lacks rest of the molecular markers of nephrons (LTL, NPHS1), which are found in BM + FGF2 + BIO pulse cultured KM spheres. Scale bar = 25 µm, n=3.

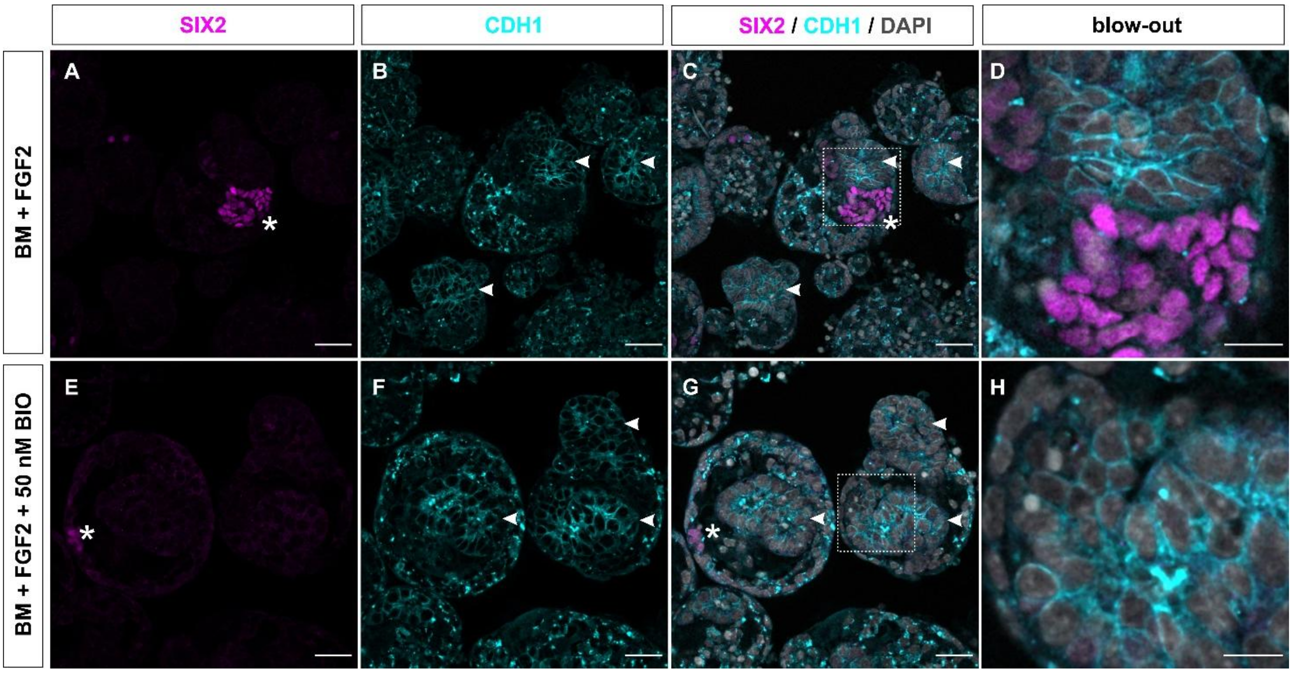

Supplemental Fig. 10. **Long-term KM spheres form CDH1 positive tubules in a comparable manner without and with low dose of BIO.** BM + FGF2 and BM + FGF2 + 50 nM BIO cultured KM spheres co-stained for nephron progenitor marker SIX2 and nephron epithelial marker CDH1 at three weeks of culture. **A** and **E** small SIX2 clusters indicated with asterisks. **B** and **F** arrowheads point to CDH1 positive nephron epithelia. **C** and **G** merged images of separate co-stained image channels, dotted line indicates region of interest containing epithelial staining. **D** and **H** blow-outs of respective regions of interest. Co-Immunostaining panel shows that in both BM + FGF2 and BM + FGF2 + constant (50 nM) BIO culture conditions progenitor cells differentiate to CDH1 positive tubules. A-C and E-G scale bar = 25 µm, D and H scale bar = 10 µm, representative images of n=3.
